# Zoonotic Endocarditis in a Man, the Netherlands 

**DOI:** 10.3201/eid2501.181029

**Published:** 2019-01

**Authors:** Janneke Sleutjens, Dennie Meijer, Paola G. Meregalli, Leendert Bakker, Jaap A. Wagenaar, Birgitta Duim, Aldert Zomer

**Affiliations:** Utrecht University, Utrecht, the Netherlands (J. Sleutjens, J.A. Wagenaar, B. Duim, A. Zomer);; Academical Medical Centre, Amsterdam, the Netherlands (D. Meijer, P.G. Meregalli);; Tergooi Hospital, Hilversum, the Netherlands (L. Bakker);; Wageningen Bioveterinary Research, Lelystad, the Netherlands (J.A. Wagenaar)

**Keywords:** zoonoses, endocarditis, horse, *Streptococcus equi* subsp. *Zooepidemicus*, whole-genome sequencing, bacteria, the Netherlands, streptococci

## Abstract

In 2017, endocarditis caused by *Streptococcus equi* subspecies z*ooepidemicus* was diagnosed in a man in the Netherlands who had daily contact with horses. Whole-genome sequencing of isolates from the man and his horses confirmed the same clone, indicating horse-to-human transmission. Systematic reporting of all zoonotic cases would help with risk assessment.

On July 23, 2017, a 62-year-old man sought care at the emergency department of Tergooi Hospital (Hilversum, the Netherlands) for general malaise and fever up to 40.6°C (105.1°F). Nine months earlier, the patient’s aortic valve had been replaced with a mechanical prosthesis. The emergency department staff found no cause for his complaints. Blood test results suggested an active infection; C-reactive protein concentration was 250 mg/L (reference value <10 mg/L). Blood culture grew gram-positive cocci, which were identified by matrix-assisted laser desorption/ionization time-of-flight mass spectrometry (Microflex LT; Bruker, http://www.bruker.com) as S*treptococcus equi* subspecies *zooepidemicus.* The MIC for penicillin was 0.016 and for gentamicin was 16 mg/L. 

Cardiac ultrasonography revealed a small, mobile structure adhering to the aortic valve prosthesis, at the side of the left ventricle outflow tract, which was possibly bacterial vegetation. An abscess was present in the aortic root at the side of the left coronary artery. The diagnosis was bacterial endocarditis of the prosthetic valve caused by *S. equi* subsp. *zooepidemicus.*

The patient received high doses of penicillin (2 million IU/4 h for 6 wk) and gentamicin (3 mg/kg/24 h for 2 wk) intravenously, according to national guidelines (https://www.swab.nl/richtlijnen). Results of subsequent blood cultures taken on days 4 and 8 after admission were negative, and the patient was discharged after 6 weeks of treatment.

Several case reports have described the potential for *S. equi* subsp. *zooepidemicus* to cause severe infection in humans. Contact with horses is a possible source of infection (*1*–*5*). The patient we describe had frequent contact with 7 horses stabled in his yard. Two weeks before his hospital admission, horse A showed signs of an upper airway infection: copious bilateral purulent nasal discharge, coughing, and fever up to 39.1°C (102.4°F; reference range 37.5°C–38.3°C [99.5°F–100.9°F]). Seventeen days later, horse B showed similar signs. After a nasal swab sample from horse B tested by the Animal Health Service (Deventer, the Netherlands) had a negative PCR result for *S. equi* subsp. *equi*, we collected nasal swab samples from all 7 horses and cultured them for streptococci on blood agar (Oxoid, http://www.oxoid.com) at 37°C for 48 h. Culture results were positive for *S. equi* subsp. *zooepidemicus* for 4 of the 7 horses. Horses A and B recovered uneventfully without antimicrobial drug treatment.

To investigate the relatedness of isolates, we fully sequenced 2 isolates from horse A, 4 isolates from horse B, 2 isolates from horse C, 1 isolate from horse D, and 1 isolate from the human patient. We identified sequence type (ST) 212 in 1 isolate from horse A, all 4 isolates from horse B, the 2 isolates from horse C, and the isolate from the patient (Appendix TableAll genomes were aligned with a selection of publicly available *S. equi* subsp. *zooepidemicus* isolates. We constructed a core-genome alignment of 1.55 Mb, representing 76% of the genome of the reference isolate MGCS10565. Comparison of the *S. equi* subsp. *zooepidemicus* core genomes showed that the human and animal ST212 isolates had 100% identical core genomes (Appendix Figure), strongly suggesting that the same clone was present in the human patient and the animals. Construction of core-genome alignments of the ST212 isolates resulted in a 1.94-Mb core genome, 97% of the genome of the ST212 isolates; only 3% of the genome was located in the accessory genome. We extracted single-nucleotide polymorphisms (SNPs) that differed between these isolates and used them to generate a minimal-spanning tree (Figure). The number of SNPs between the human and horse isolates did not differ significantly from the number of SNPs differing between horse isolates, thereby demonstrating animal-to-human transmission of *S. equi* subsp. *zooepidemicus*.

**Figure:**
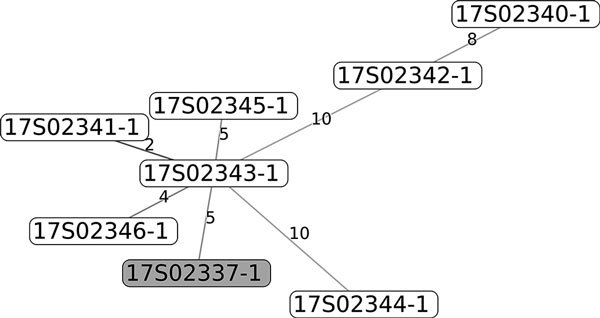
Minimal-spanning tree of 34 single-nucleotide polymorphisms. Gray indicates the human isolate. Single-nucleotide polymorphisms counts are given.

In healthy horses, *S. equi* subsp. *zooepidemicus* is a commensal organism of the upper respiratory and lower genital tracts and can cause secondary infections (*6*). However, in 2010, a large outbreak in Iceland showed that *S. equi* subsp. *zooepidemicus* might be a primary pathogen that spreads clinically among horses without any other predisposing factors (*7*). Infections with *S. equi* subsp. *zooepidemicus* in humans, especially confirmed cases originating from contact with horses, are rare. Only a few reports confirm a horse as the source of infection by whole-genome sequencing (*7*,*8*). Systematic reporting of suspected or confirmed transmission of pathogens between horses and humans is lacking. Such reporting would support the estimation of the burden of equine-origin zoonotic infections in humans, which is needed as the equine industry continues to grow. Collaboration among disciplines to develop such a reporting system is fundamental for enabling reliable assessment of the potential risk for humans to become ill after contact with horses and the usefulness of implementing precautionary measures for patients with specific conditions.

Appendix Supplementary methods and results from investigation of zoonotic endocarditis in the Netherlands.

## References

[1] Eyre DW, Kenkre JS, Bowler ICJW, McBride SJ. *Streptococcus equi* subspecies *zooepidemicus* meningitis—a case report and review of the literature. Eur J Clin Microbiol Infect Dis. 2010;29:1459–63. 10.1007/s10096-010-1037-5 20820836

[2] Minces LR, Brown PJ, Veldkamp PJ. Human meningitis from *Streptococcus equi* subsp. z*ooepidemicus* acquired as zoonoses. Epidemiol Infect. 2011;139:406–10. 10.1017/S0950268810001184 20492747

[3] Altreuther M, Lange C, Myhre HO, Hannula R. Aortic graft infection and mycotic aneurysm with *Streptococcus equi zooepidemicus*: two cases with favorable outcome of antibiotic treatment. Vascular. 2013;21:6–9. 10.1258/vasc.2011.cr0299 22375044

[4] van Samkar A, Brouwer MC, van der Ende A, van de Beek D. *Streptococcus equi* meningitis. Clin Microbiol Infect. 2016;22:e3–4. 10.1016/j.cmi.2015.09.003 26369601

[5] Kittang BR, Pettersen VK, Oppegaard O, Skutlaberg DH, Dale H, Wiker HG, et al. Zoonotic necrotizing myositis caused by *Streptococcus equi* subsp. z*ooepidemicus* in a farmer. BMC Infect Dis. 2017;17:147. 10.1186/s12879-017-2262-7 28201995 PMC5312586

[6] Fulde M, Valentin-Weigand P. Epidemiology and pathogenicity of zoonotic streptococci. In: Chhatwal G, editor. Current Topics in Microbiology and Immunology. New York: Springer;2012. p. 49–81. 10.1007/82_2012_277 23192319

[7] Björnsdóttir S, Harris SR, Svansson V, Gunnarsson E, Sigurðardóttir ÓG, Gammeljord K, et al. Genomic dissection of an Icelandic epidemic of respiratory disease in horses and associated zoonotic cases. MBio. 2017;8:e00826–17. 10.1128/mBio.00826-17 28765219 PMC5539424

[8] Pelkonen S, Lindahl SB, Suomala P, Karhukorpi J, Vuorinen S, Koivula I, et al. Transmission of *Streptococcus equi* subspecies *zooepidemicus* infection from horses to humans. Emerg Infect Dis. 2013;19:1041–8. 10.3201/eid1907.121365 23777752 PMC3713971

