# Supplementary material for: Zoonotic Endocarditis in a Man, the Netherlands
Source: Emerg Infect Dis. 2019 Jan;25(1):180–2. doi: 10.3201/eid2501.181029 (PMC6302571; doi:10.3201/eid2501.181029)
Supplement: Appendix — Supplementary methods and results from investigation of zoonotic endocarditis in the Netherlands. [file 18-1029-Techapp-s1.pdf]

Article DOI: https://doi.org/10.3201/eid2501.181029

Zoonotic Endocarditis in a Man, the Netherlands

Appendix

Supplementary Methods

DNA was isolated using the MO-BIO Ultra Clean Microbial DNA isolation kit (MO-BIO Laboratories INC, Carlsbad, CA). Sequencing was performed using Illumina NextSeq with 150 bp paired end reads, that were assembled using SPAdes 3.10.1 (*1*). The average coverage was 90x. The quality of genomes obtained in this study and the downloaded genomes was assessed with CheckM (*2*). Only genomes with >98% completeness score were included. The whole genome sequence data of the isolates have been deposited at the Short Read Archive under project PRJEB27317 with the accession numbers listed in the Appendix Table. A core-genome alignment using Parsnp v1.2 (*3*) on contigs larger than 2 kbp was performed to construct phylogenetic maximum likelihood trees using FastTree2 (*4*) and visualized with FigTree (http://tree.bio.ed.ac.uk/software/figtree/). Genomes were annotated with Prokka (*5*) and orthology was determined using Roary (*6*).

References

1. Bankevich A, Nurk S, Antipov D, Gurevich AA, Dvorkin M, Kulikov AS, et al. SPAdes: a new genome assembly algorithm and its applications to single-cell sequencing. J Comput Biol. 2012;19:455–77. PubMed http://dx.doi.org/10.1089/cmb.2012.0021

2. Parks DH, Imelfort M, Skennerton CT, Hugenholtz P, Tyson GW. CheckM: assessing the quality of microbial genomes recovered from isolates, single cells, and metagenomes. Genome Res. 2015;25:1043–55. PubMed http://dx.doi.org/10.1101/gr.186072.114

3. Treangen TJ, Ondov BD, Koren S, Phillippy AM. The Harvest suite for rapid core-genome alignment and visualization of thousands of intraspecific microbial genomes. Genome Biol. 2014;15:524. PubMed http://dx.doi.org/10.1186/s13059-014-0524-x

4. Price MN, Dehal PS, Arkin AP. FastTree 2 – Approximately Maximum-Likelihood Trees for Large Alignments. Poon AFY, editor. PLoS One. 2010 Mar 10;5(3):e9490.

5. Seemann T. Prokka: rapid prokaryotic genome annotation. Bioinformatics. 2014;30:2068–9. PubMed http://dx.doi.org/10.1093/bioinformatics/btu153

6. Page AJ, Cummins CA, Hunt M, Wong VK, Reuter S, Holden MTG, et al. Roary: rapid large-scale prokaryote pan genome analysis. Bioinformatics. 2015;31:3691–3. PubMed http://dx.doi.org/10.1093/bioinformatics/btv421

Appendix Table. *Strep. Equi.* subsp. *Zooepidemicus* strains investigated by whole genome sequencing

Strain	Source	MLST type	Coverage	Accession	Reference	Assembly
17S02337–1	human	212	85	ERS2550930		
17S02338–1	horse D	92	83	ERS2550931		
17S02339–1	horse A	99	100	ERS2550932		
17S02340–1	horse A	212	95	ERS2550933		
17S02341–1	horse B	212	99	ERS2550934		
17S02342–1	horse B	212	92	ERS2550935		
17S02343–1	horse B	212	85	ERS2550936		
17S02344–1	horse B	212	68	ERS2550937		
17S02345–1	horse C	212	69	ERS2550938		
17S02346–1	horse C	212	95	ERS2550939		
MGCS10565		72		CP001129.1	ref	GCA_000020765.1_ASM2076v1
H70		1		FM204884.1	ref	GCA_000026605.1_ASM2660v1
BHS5		123		CABY01000011.1	ref	GCA_000208725.1_ASM20872v1
ATCC35246		194		CP002904.1	ref	GCA_000219765.1_ASM21976v1
SzS31A1		279		AUXA02000089.1	ref	GCA_000445225.2_ASM44522v2
CY		194		CP006770.1	ref	GCA_000696505.1_ASM69650v1
2329		-		JTJH01000001.1	ref	GCA_000836615.1_ASM83661v1
Sz105		140		JATZ01000041.1	ref	GCA_000876195.1_ASM87619v1
Sz4is		279		JAUE01000033.1	ref	GCA_000876215.1_ASM87621v1
SzAM35		65		JATY01000119.1	ref	GCA_000876275.1_ASM87627v1
SzAM60		-		JATX01000052.1	ref	GCA_000876285.1_ASM87628v1
Sz16		156		JATW01000074.1	ref	GCA_000876295.1_ASM87629v1
Sz12is		279		JAUD01000037.1	ref	GCA_000876305.1_ASM87630v1
Sz5		303		JAUC01000036.1	ref	GCA_000876355.1_ASM87635v1
Sz35		203		JAUB01000112.1	ref	GCA_000876365.1_ASM87636v1
Sz57		96		JAUA01000048.1	ref	GCA_000876375.1_ASM87637v1

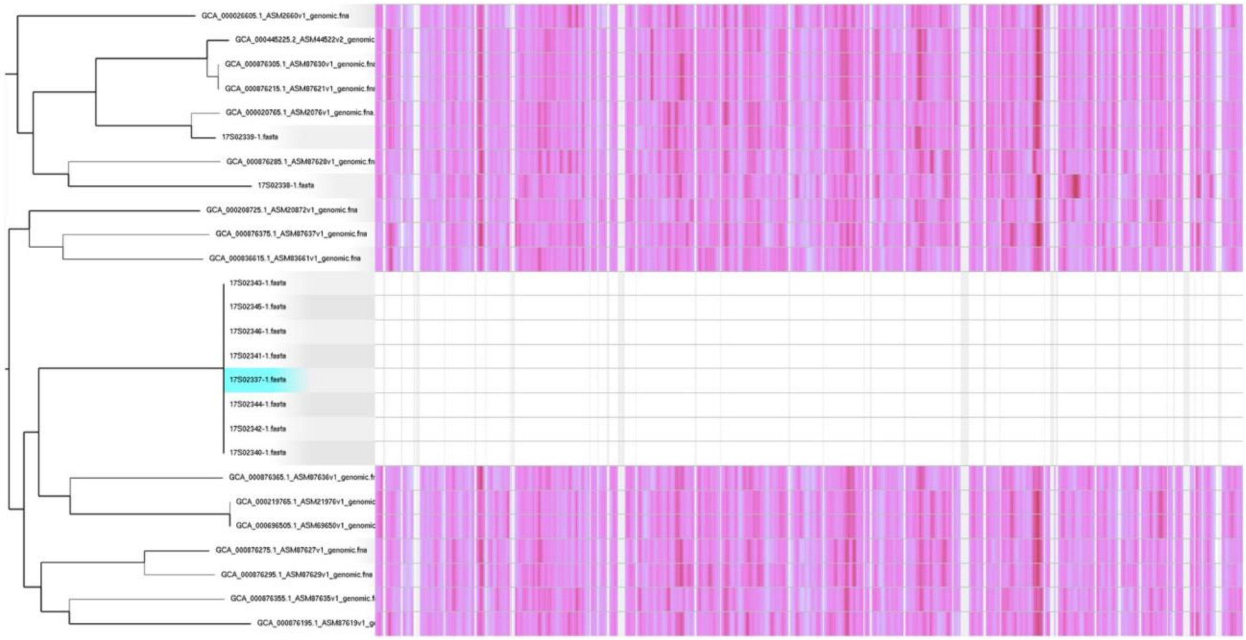

Appendix Figure. Phylogenetic tree and single-nucleotide polymorphism locations of whole-genome alignment data of human, horse, and reference isolates. Blue shading, human isolate; purple, single-nucleotide polymorphisms; gray, DNA regions that were excluded from the analysis.
